# The dynamic cycle of bacterial translation initiation factor IF3

**DOI:** 10.1093/nar/gkab522

**Published:** 2021-06-23

**Authors:** Jose A Nakamoto, Wilfredo Evangelista, Daria S Vinogradova, Andrey L Konevega, Roberto Spurio, Attilio Fabbretti, Pohl Milón

**Affiliations:** Laboratory of Applied Biophysics and Biochemistry, Centre for Research and Innovation, Health Sciences Faculty, Universidad Peruana de Ciencias Aplicadas (UPC), Lima 15023, Peru; Laboratory of Applied Biophysics and Biochemistry, Centre for Research and Innovation, Health Sciences Faculty, Universidad Peruana de Ciencias Aplicadas (UPC), Lima 15023, Peru; Petersburg Nuclear Physics Institute, NRC ‘Kurchatov Institute’, Gatchina 188300, Russia; NanoTemper Technologies Rus, Saint Petersburg 191167, Russia; Petersburg Nuclear Physics Institute, NRC ‘Kurchatov Institute’, Gatchina 188300, Russia; NRC ‘Kurchatov Institute’, Moscow 123182, Russia; Peter the Great St. Petersburg Polytechnic University, Saint Petersburg 195251, Russia; Laboratory of Genetics, School of Biosciences and Veterinary Medicine, University of Camerino, Camerino 62032, Italy; Laboratory of Genetics, School of Biosciences and Veterinary Medicine, University of Camerino, Camerino 62032, Italy; Laboratory of Applied Biophysics and Biochemistry, Centre for Research and Innovation, Health Sciences Faculty, Universidad Peruana de Ciencias Aplicadas (UPC), Lima 15023, Peru

## Abstract

Initiation factor IF3 is an essential protein that enhances the fidelity and speed of bacterial mRNA translation initiation. Here, we describe the dynamic interplay between IF3 domains and their alternative binding sites using pre-steady state kinetics combined with molecular modelling of available structures of initiation complexes. Our results show that IF3 accommodates its domains at velocities ranging over two orders of magnitude, responding to the binding of each 30S ligand. IF1 and IF2 promote IF3 compaction and the movement of the C-terminal domain (IF3C) towards the P site. Concomitantly, the N-terminal domain (IF3N) creates a pocket ready to accept the initiator tRNA. Selection of the initiator tRNA is accompanied by a transient accommodation of IF3N towards the 30S platform. Decoding of the mRNA start codon displaces IF3C away from the P site and rate limits translation initiation. 70S initiation complex formation brings IF3 domains in close proximity to each other prior to dissociation and recycling of the factor for a new round of translation initiation. Altogether, our results describe the kinetic spectrum of IF3 movements and highlight functional transitions of the factor that ensure accurate mRNA translation initiation.

## INTRODUCTION

**Figure 1.**
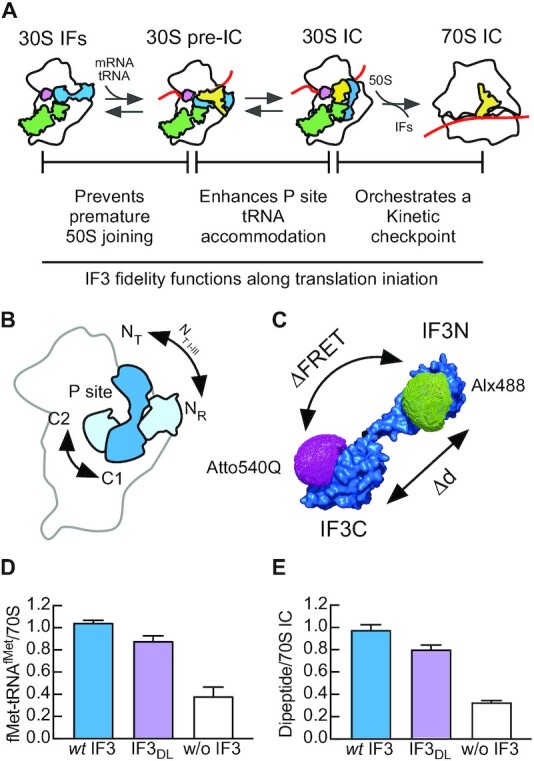
Double labelled Initiation factor IF3 (IF3_DL_) and the progression of bacterial translation initiation. (**A**) Scheme of the main translation initiation intermediates, 30S-bound Initiation Factors (IFs) (30S–IFs), the 30S pre-IC, the 30S IC, and the ready to elongate 70S IC. IF1 and IF2 are schematically represented in pink and green, respectively. The initiator tRNA is yellow and the red line indicates the mRNA. The main fidelity functions of IF3 (blue) are indicated. (**B**) Diagram of the 30S-bound IF3 indicating the alternative binding sites for each IF3 domain as observed in structural studies (22–24). IF3C can bind to either the C1 or C2 binding sites and IF3N can either bind the 30S platform near uS11 (N_R_) or move towards the P site, ready to interact with initiator tRNA (N_T_). IF3N interacting with fMet-tRNA^fMet^ was shown to fluctuate along three intermediates (N_T-I_ to N_T-III_, Supplementary Table S1) (22). Black arrows indicate the directionality of IF3 domain movements. (**C**) Structural representation of IF3_DL_ used in this work to monitor intramolecular Föster Resonance Energy Transfer (FRET). Changes in FRET allows to monitor distance changes between IF3 domains (36). Green and magenta dots represent accessible volumes of donor (Alexa 488) and acceptor (Atto 540Q) FRET pairs, specifically linked to IF3N and IF3C, respectively. (**D**) Activity of IF3_DL_ (purple) as compared to *wt* IF3 (blue) and in the absence of the factor (white). fMet-tRNA^fMet^ binding to 70S complexes was measured by HPLC using isocratic flow on a SEC BioSuite 450HR column (Waters, Milford, MA) for macromolecular complexes (Supplementary Figure S2A–S2C). The ratio between fMet-tRNA^fMet^ and 70S ribosomes coeluting together was used to calculate 70S IC formation efficiency. Error bars correspond to standard errors of the mean. (**E**) Comparison of dipeptide formation efficiency as calculated from Equation (6) was measured as a function of IF3 using HPLC and liquid scintillation radioactivity counting of [^14^C]-Val-tRNA^Val^ (Supplementary Figure S2D–S2F).

IF3 is an essential bacterial protein consisting of two domains (IF3C and IF3N) separated by a hydrophilic, lysine-rich, linker (1–3). IF3 is involved in all steps of the bacterial translation initiation driving the 30S ribosomal subunit trough the transition from the 30S initiation complex (30S IC) to a productive 70S initiation complex (70S IC) (4–7). When bound to the 30S subunit, IF3 prevents the premature 50S subunit association and increases the rate of the P site codon-anticodon interaction between fMet-tRNA^fMet^ and the initiation triplet of the mRNA (5,8,9) (Figure 1A). As a direct consequence, IF3 acts as an initiation fidelity factor by increasing the dissociation rate of non-canonical and pseudo-30S initiation complexes (8,10,11). In late events of translation initiation, IF3 orchestrates a kinetic checkpoint defining the rate at which the initiating ribosome enters the elongation phase of protein synthesis (5).

Although IF3 functions during the initiation phase of protein synthesis are well established, dynamic aspects of IF3 domains and their role in translation initiation fidelity were not fully investigated. Furthermore, dynamic aspects of IF3 bound to ribosomal subunits and the release of the factor after the formation of a productive 70S IC are still debated (12,13). A model where IF3C and IF3N domains independently bind the 30S ribosomal subunit was initially proposed in the early ‘80s (14) and then confirmed by NMR analysis and time-resolved chemical probing experiments (15,16). Several studies have dealt with the assignment of a topographical localization of IF3 on the 30S ribosomal subunit, producing conflicting conclusions (17–20). More recently, CryoEM and single-molecule FRET experiments demonstrated that IF3 undergoes large conformational changes along the initiation pathway (21,22). CryoEM reconstructions, together with other recent structural studies, contributed to the identification of a minimum of four different arrangements of IF3 on the 30S ribosomal subunit and two alternative binding sites for each domain of the factor (22–24) (Figure 1B). IF3C can interact with two distinct binding sites on the 30S subunit, either at the P site and in contact with IF1 (C2 position in Figure 1B), or at helix 45 and helix 24, away from both IF1 and the P site (C1 position in Figure 1B). Similarly, IF3N can occupy two binding sites, either on the 30S platform near uS11, or on the elbow of tRNA^fMet^, herein called N_R_ and N_T_, respectively (Figure 1B). Despite these detailed structural data, a fully comprehensive dynamic correlation between IF3 interaction sites, the ligands present on the 30S subunit and the fidelity functions of the factor remained elusive.

In this study, we use a combination of Föster Resonance Energy Transfer (FRET) and pre-steady state kinetics techniques combined with molecular modelling to monitor IF3 movements on the ribosome and to construct a comprehensive model depicting the dynamics of IF3 domains along the cycle of translation initiation, from the initial binding until the recycling of the factor.

## MATERIALS AND METHODS

### *Escherichia coli* strains, expression vectors, cell growth and protein expression

Expression vectors pET24c containing *InfA*, *InfB*, *InfC* and *InfC-E166C* were acquired commercially (GenScript, Piscataway, NJ, USA). Competent *E. coli* BL21 (DE3) cells were CaCl_2_ transformed (Mix & Go, ZymoResearch, Irvine, CA, USA) with expression vectors coding for IF1, IF2 with an NTD His-tag, IF3 or IF3_E166C_. Typically, 2 L of Luria Bertani (LB) medium were used to grow the BL21 (DE3) strains to an OD_600 nm_ of 0.5. Protein expression was induced by adding 1 mM Isopropyl β-d-1-thiogalactopyranoside (IPTG, ThermoScientific, Waltham, MA, USA). Cells were allowed to express the factors for 3 hours prior to harvesting by centrifugation at 5000 × g at 4°C. Cells were lysed by sonication (20 cycles of 10 s followed by 30 s without sonication) in Buffer A (50 mM HEPES (pH 7.1), 100 mM NH_4_Cl, 10 mM MgCl_2_, 10% glycerol, 6 mM 2-mercaptoethanol) for IF1 and IF3; or Buffer B (50 mM sodium phosphate buffer (pH 7.7), 300 mM NaCl, 30 mM imidazole) for IF2. Debris and supernatant were separated by centrifugation at 15 000 × g for 30 min a 4°C.

### Purification of initiation factors

Initiation factors IF1 and IF3 were purified by Cation exchange chromatography on a HiTrap™ SP HP column (GE Healthcare Life Sciences, Chicago, IL, USA). Cleared lysates were loaded onto the column (1 ml column volume, CV) and subsequently subjected to a linear NH_4_Cl gradient (0.05–1 M) in Buffer A using a HPLC system (Jasco, Hachiōji, Tokyo, Japan). IF3 and IF1 eluted at 700 mM and 400 mM NH_4_Cl, respectively. Best separation conditions were found at 1 ml/min flow rate and 20 CVs long gradient, collecting fractions of 1 ml each. Protein elution was followed by absorbance at 290 nm and SDS-Polyacrylamide Gel Electrophoresis (SDS-PAGE, 15%). While IF3 eluted with an elevated degree of purity, IF1 fractions contained high molecular weight contaminants. Protein contaminants were eliminated by subjecting the combined IF1 fractions to Amicon® Ultra 30K Da centrifugal filters (Merck, Kenilworth, NJ, USA) followed by concentration on a HiTrap™ SP HP (GE Healthcare Life Sciences, Chicago, IL, USA), single step eluted with 1 M NH_4_Cl in Buffer A. Eluted proteins were dialyzed against Storage buffer (50 mM Tris–HCl (pH 7.1), 200 mM NH_4_Cl, 10% Glycerol, 6 mM 2-mercaptoethanol) and 50 μl aliquots were stored at –80°C. Purity was assayed by 15% SDS-PAGE and total protein staining using blue Coomassie. Initiation factor IF2 was purified by affinity chromatography on a Histrap FF crude column (1 ml) (GE Healthcare Life Sciences, Chicago, IL, USA). Supernatants were manually loaded to the column, followed by 5 ml of wash buffer (50 mM Imidazole in Buffer B) and to 2.5 ml of elution buffer (250 mM Imidazole in Buffer B). The first 1.5 ml were collected and subjected to dialysis in Buffer A. Finally, IF2 was further purified by Cation exchange chromatography as explained above for IF1 and IF3.

### Double labelling of IF3

IF3_E166C_ was subjected to extensive dialysis in Labelling buffer (50 mM HEPES (pH 7.1), 100 mM NH_4_Cl, 10% glycerol and 0.5 mM TCEP) in a D-Tube™ Dialyzer Maxi (Merck, Kenilworth, NJ, USA) to remove traces of 2-mercaptoethanol as the reducing agent strongly inhibits the coupling to maleimide linked dyes to cysteines. First, the C-terminal was labelled at the recombinant cysteine (C166) as it is exposed and efficiently reacts with maleimide derivatives. IF3_E166C_ with a 10-fold excess of Atto-540Q maleimide (Atto-Tec GmbH, Siegen, Germany) was incubated in Labelling buffer for 20 min. The reaction was stopped by the addition of 6 mM 2-mercaptoethanol. The modified IF3, termed IF3_C540Q_, was purified from unreacted dyes on a HiTrap SP HP column (GE Healthcare Life Sciences, Chicago, Il, USA) as described above. After 10 CV washes with Buffer A containing 100 mM NH_4_Cl, a single step elution was applied using 3 ml of 1 M NH_4_Cl in Buffer A. Typically, elution of the labelled protein is readily visible in the first 0.5 ml fraction. IF3_C540Q_ was subsequently dialyzed as mentioned above in labelling buffer containing 2 M UREA. The partial denaturation of IF3 results in the exposure of the otherwise buried cysteine at position 65 of the NTD. The unfolded protein was incubated with a 20-fold molar excess of Alexa488 maleimide (ThermoScientific, Waltham, MA, USA) for 1 h at RT, under mild shacking. IF3_C540Q–N488_ (IF3_DL_) was purified from the unreacted dye as described above using HiTrap SP HP column (GE Healthcare Life Sciences, Chicago, IL, USA). Eluted proteins were dialyzed against Storage buffer and 20 μl aliquots were stored at –80°C. Purity and efficiency of labelling was assayed by 15% SDS-PAGE where fluorescence was observed under a UV trans-illuminator and total protein was detected by Blue Coomassie staining.

### Ribosomal subunits, fMet-tRNA^fMet^ and mRNAs

30S and 50S ribosomal subunits were purified from tight coupled 70S ribosomes by sucrose gradient centrifugation under dissociative conditions (3.5 mM MgCl_2_), essentially as described (25). 30S subunits were reactivated by 20 mM MgCl_2_ treatment for 30 min at 37°C prior to use. fMet-tRNA^fMet^ was *in vitro* aminoacylated, formylated and purified by reverse phase HPLC as described (25). The model mRNAs were chemically synthetized by Trilink (San Diego, CA, USA) or Microsynth (Balgach, Switzerland) with the following sequence: 5′-AAA CAA UUG GAG GAA UAA GGU (aug/auu/acg) UUU GGC GGA AAA CGA. 70S IC formation and dipeptide analysis used the *in vitro* transcribed mMVF mRNA: 5′-AAG UUA ACA GGU AUA CAU ACU aug GUU UUU AUU ACU ACG. Alternative initiation codons are shown in lower case in parentheses.

### Stopped–flow measurements and analysis

Fluorescence stopped-flow measurements were performed using a SF-300X stopped-flow apparatus (KintekCorp, Snowshoe, PA, USA) or a SX20 (Applied Photophysics, Leatherhead, Surrey, UK) by rapidly mixing equal volumes of each reacting solution. The excitation wavelength for Alexa 488 was 470 nm. The emission signal was measured after a long-pass optical filter with a 515 nm cut-off. 1000 points were acquired in each measurement. 7 to 10 replicates were recorded for each reaction and subsequently averaged. All stopped flow reactions were performed in TAKM_10_ buffer (50 mM Tris (pH 7.5), 70mM NH_4_Cl, 30 mM KCl, 10 mM MgCl_2_, 6 mM 2-Mercaptoethanol) at 25°C. Unless otherwise stated, 30S complexes were prepared by mixing 0.1 μM 30S, 0.08 μM IF3_DL_, 0.3 μM IF1, 0.3 μM IF2, 100 μM GTP, 0.5 μM mRNA and/or fMet-tRNA^fMet^. Mixtures were incubated at 37°C for 30 min and centrifuged at 14 000 × g for 5 min prior to the measurement. Fluorescence time courses were baseline removed, and to highlight the overall conservation of the signal along the full cycle of IF3, an amplitude factor corresponding to the previous reaction was added. All graphical representations have the same Y axis range and represent changes of Volts as measured by the Instrument Photomultiplier. Time courses were analysed by non-linear regression with one or two exponential terms (Equation 1) using the Prism Software v. 9.0 (Graphpad, San Diego, CA, USA).

(1) $$\begin{equation*}F = {F_0} + \mathop \sum \limits_{i = 1}^n {F_i}{e^{ - {k_{appi}}t}}\end{equation*}$$

With $t$ being the time; ${k_{appi}}$, an apparent rate constant for a phase *i*; $F$, the fluorescence; ${F_0}$, the initial fluorescence value; ${F_i}$, the fluorescence change amplitude related to ${k_{appi}}$; and $n$, the number of phases of the kinetic scheme describing partial reactions.

30S, IF2, IF1, and mRNA showed conformational changes of IF3_DL_ slower than their respective bimolecular reactions (4), allowing to fit the obtained rates as a function of titrant concentration with a hyperbolic equation (Equation 2) where ${V_{max}}$ and ${K_s}$ could be calculated.

(2) $$\begin{equation*}Y = \frac{{{V_{max}}\left[ C \right]}}{{{K_s} + \left[ C \right]}}\ \end{equation*}$$

With $Y$ being the reaction rate; $[ C ]$, the concentration; *V_max_*, the maximum rate; and *K_S_*, the rapid equilibration constant.

(3) $$\begin{equation*}{k_{AV}} = \frac{{\mathop \sum \nolimits_{i = 1}^n {F_i}{k_{appi}}}}{{\mathop \sum \nolimits_{i = 1}^n {F_1}}}\ \end{equation*}$$

With ${k_{AV}}$ being the averaged apparent rate constant calculated from Equation (1). Standard errors of ${k_{AV}}$ were calculated according to error propagation functions for addition and product using the mean and standard deviation of each term of Equation (3).

(4) $$\begin{eqnarray*} {\sigma _{{K_{AV}}}} &=& {K_{AV}} *\left( {{\left( {\frac{{{{\left( {\mathop \sum \nolimits_{i = 1}^n {{\left( {{{\left( {\left( {{{\left( {\frac{{{\sigma _{{F_i}}}}}{{{F_i}}}} \right)}^2} + {{\left( {\frac{{{\sigma _{{k_{appi}}}}}}{{{k_{appi}}}}} \right)}^2}} \right)*{F_i}*{k_{appi}}} \right)}^{\frac{1}{2}}}} \right)}^2}} \right)}^{\frac{1}{2}}}}}{{\mathop \sum \nolimits_{i = 1}^n \left( {{F_i}*{k_{appi}}} \right)}}} \right)}^2} \right. \nonumber \\ && \left. + \, {{\left( {\frac{{{{\left( {\mathop \sum \nolimits_{i = 1}^n {\sigma _{{F_i}}}^2} \right)}^{\frac{1}{2}}}}}{{\mathop \sum \nolimits_{i = 1}^n {F_i}}}} \right)}^2} \right)^{\frac{1}{2}} \end{eqnarray*}$$

With ${K_{AV}}$ being the average rate constant calculated from Equation (3); $n$, the number of phases in the reaction; ${F_i}$, the fluorescent change related to the $i$ phase; ${\sigma _{{F_i}}}$, the standard deviation corresponding to ${F_i}$; ${k_{appi}}$, the apparent rate constant of phase $i$; and ${\sigma _{{k_{appi}}}}$, the standard deviation corresponding to ${k_{appi}}$.

### 70S IC and dipeptide formation

The efficiency of fMet-tRNA^fMet^ (Flu) (25) binding to 70S ribosomes to build a 70S IC was measured by HPLC under isocratic flow in TAKM_7_ buffer (50 mM Tris (pH 7.5), 70 mM NH_4_Cl, 30 mM KCl, 7 mM MgCl_2_, 6 mM 2-mercaptoethanol) using the BioSuite 450HR size exclusion column (Waters, Milford, MA) for macromolecular complexes. tRNA fluorescence was excited at 460 nm and measured at 540 nm. 70S absorbance was measured at 260 nm. Coelution of fMet-tRNA^fMet^ (Flu) and 70S ribosomes was assigned to 70S IC formation. The efficiency of complex formation was calculated from the areas under the peaks corresponding to 70S ICs and unbound tRNA as follows:

(5) $$\begin{equation*}{E_{IC}} = \left( {\frac{{Ab}}{{Ab + Au}}} \right)\ \left( {\frac{{\left[ {tRNA\left( {Flu} \right)} \right]}}{{\left[ {70S} \right]}}} \right)\end{equation*}$$

with Ab = bound area, Au = unbound area. To analyse dipeptide formation fMet-[14C]-Val, 0.1 μM 70S ICs complexes, containing fMet-tRNA^fMet^ were formed in the absence or presence of IF3 or IF3_DL_. Then, a mixture of ternary complex EF-Tu·GTP·[^14^C]-Val-tRNA^Val^ (0.2 μM) was added to the initiation complexes. The experiments were performed in TAKM_7_ buffer and 1 mM GTP at 25°C and incubated for 1 min. Reactions were quenched with 1/10 volume of 5 M KOH and hydrolysed for 30 min at 37°C. Samples were neutralized with 1/10 volume of glacial acetic acid and analysed by reversed-phase HPLC and liquid scintillation counting as described before (26). Dipeptide efficiency was calculated from the amounts of [^14^C]-Val corresponding to fMet- [^14^C]-Val dipeptides and unreacted [^14^C]-Val as follows:

(6) $$\begin{eqnarray*} {E_{Dip}} &=& \left( {\frac{{fMet\left[ {14C} \right]Val}}{{fMet\left[ {14C} \right]Val + \left[ {14C} \right]Val}}} \right) \nonumber \\ &&\times\, \left( {\frac{{\left[ {EF\_Tu\ TC} \right]}}{{\left[ {70S} \right]}}} \right) \end{eqnarray*}$$

### Structural modelling of the 30S-bound IF3_DL_ and interdomain distance analysis

Structures of the 30S pre-IC and 30S IC were obtained from the Protein Data Bank (PDB: 5lmn, 5lms, 5lmt, 5lmu, 5lmv) (22). IF3 was extracted into new PDB files from each complex using Chimera software (27). IF3_E166C_ was then modelled with the Swiss Modeler server using each IF3 PDB file as template (28). Finally, the modelled IF3_E166C_ replaced the original IF3 in the reported 30S IC structures. fMet-tRNA^fMet^ was removed from all PDB files to generate a subset of structures lacking the tRNA. The accessible volume (AV) of each fluorophore on the 30S ICs structures was determined with FRET-restrained Positioning and Screening software (Supplementary Tables S1 and S2) (29). The fluorophore dimension parameters were: linker length: 15 Å; Width: 4.5 Å; Dye radius: 4.5 Å as defined from a model generated with Maestro software (Schrödinger, NY, USA). The distances between points of the AV of donor and acceptor dyes were calculated in Matlab (Mathworks, Natick, MA, USA) using an analytical geometry approximation. The distance distribution between AVs was determined with Prism 9.0 (Graphpad, San Diego, CA, USA).

### Molecular Dynamics of free IF3

The full structure of *E. coli wt* IF3 was modelled using Modeller v.9.25 (30,31) and four template structures from three different organisms: *Thermus thermophilus*, PDB id 5LMN:X; *Geobacillus stearothermophilus*, PDB id: 1TIF, *E. coli*, PDB id: 2IFE and 5ME0:Z, with the sequences previously aligned using ClustalW v. 2.1 and edited manually in cases where it was necessary. The obtained *wt* model was used to build the E166C using Pymol v1.8 (Delano W.L. & Schrödinger, 2009), and to build the IF3 model with Alexa 488 and Cy5 fluorophores following the procedure described by Schepers and Gohlke. (32). Cy5 was used for these simulations because the chemical structure of Atto540Q is not available. Three systems were constructed for MD simulations and subjected to energy minimization. Briefly, the IF3 structure was explicitly solvated in a TIP3P water in a cubic box of 8 nm × 8 nm × 8 nm. Periodic boundary conditions in all directions were applied with electrostatic type fast smooth particle mesh Ewald. A 50 000-step energy minimization was performed using the steepest decent algorithm and maximum force equal to 10 kJ mol^−1^ nm^−1^. Molecular dynamics simulations were carried out using the Gromacs v2018.1 (33,34) simulation engine and the AMBER-f99sb force field (35). For each species, a constant-volume and constant-temperature (NVT) 100 ps equilibration with a 2 fs integration time step was performed, then a 100 ps equilibration phase was conducted under constant-pressure thermodynamic ensemble and 1.0 bar pressure conditions using the Berendsen coupling. Finally, a 100 ns production was ran using the Parrinello−Rahman algorithm. Atomic coordinates were saved on a disk every 10 ps. Then, conformations from arbitrary states of *wt* IF3 were selected for additional 100 ns of MD simulations. Distance between α-Carbon atoms of residues 65 and 166 was used as conformation coordinate to measure protein compactness for each trajectory of the systems analysed here.

## RESULTS

### Experimental approach

To monitor the conformational rearrangements of IF3, we used a fluorescent derivative of the factor harbouring donor and silent acceptor dyes specifically linked to the N and C terminal domains, respectively (Figure 1C) (36). The design of the double-labelled IF3 (IF3_DL_) involved minimal mutagenesis of the factor, which was modified at a single amino acid position (E166C). The cysteine in IF3_E166C_ is solvent exposed for the free or 30S-bound IF3, does not disturb any secondary structure of IF3C, and was used previously for FRET-based kinetics (4,5,12,13,25,37). On the other hand, the *wt* cysteine at position 65 was used with fluorophores that have five carbon linkers to reduce the potential interference of the dye with the IF3N structure. The resulting IF3_DL_ allows to monitor intramolecular FRET changes resulting from the movement of IF3 domains with respect to each other. Donor fluorescence values were used to measure FRET changes of IF3_DL_ in a stopped-flow apparatus, allowing to monitor IF3 movements in real-time. An increase in time of donor fluorescence indicates that IF3 domains are moving apart and, *vice versa*, a decrease of the observed fluorescence indicates that the domains are getting close to each other (Supplementary Figure S1).

Biochemical characterization of IF3_DL_ showed that the fluorescent derivative promoted 70S IC formation similarly to *wt* IF3 (Figure 1D). Furthermore, IF3_DL_ activity was retained in multiple turnover conditions for the factor, i.e. 1:10 ratio towards 70S ribosomes, where IF3 must perform all its function 10-times to promote maximal 70S IC formation (Supplementary Figure S2). To investigate if the resulting 70S ICs were capable of entering the elongation phase, we measured dipeptide formation. IF3_DL_ promoted the formation of in-frame 70S elongating complexes similarly to *wt* IF3 (Figure 1E). Furthermore, Molecular Dynamics showed that neither the labelled position 65 nor the introduced cysteine at position 166 altered the structures of their respective domains (Supplementary Figure S1D,E). Altogether, the biochemical and structural characterization of IF3_DL_ indicates that the labelled factor is functional along the complete translation initiation cycle.

To position each domain of IF3_DL_ on the 30S subunit, we first modelled the labelled factor by homology and used FRET-restrained Positioning and Screening software to model the accessible volumes of the dyes on each IF3 domain at all possible binding combinations on the 30S subunit (Supplementary Figures S3 and S4) (see Materials and Methods). The resulting accessible volumes for the dyes allowed us to calculate the theoretical distances between fluorophores and domains (Supplementary Figure S3). To relate the distance changes to the positioning of each domain, we assigned the largest and shortest domain distances to the minimal and maximal FRET states, respectively, as measured along all IF3_DL_-dependent reactions (Supplementary Table S1). The most extended layout of IF3_DL_ was found when the factor binds to C1 and N_R_ positions, which are not available in high-resolution cryoEM reconstructions. The most compact states of IF3 were found for IF3C occupying the C2 site while IF3N positioned at the N_TII_, corresponding to a 30S pre-IC, or N_TIII_ sites (Supplementary Table S1) (22). Intermediate distances and the resulting proposed positioning are suggested considering the known relation between IF3 and the directionality of the interdomain distance change corresponding the ligand that was investigated. Thus, the structural analysis of IF3_DL_ bound to 30S IC intermediates provides all conformational states of the factor and can serve as a guide to allocate potential positioning of each domain together with their corresponding dynamics.

### IF3 domains rapidly adopt an extended layout on the 30S

**Figure 2.**
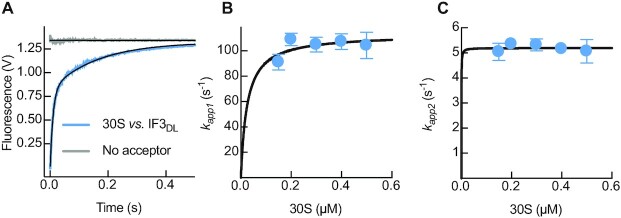
IF3_DL_ binds the 30S subunit rapidly acquiring an elongated layout. (**A**) Time course of donor fluorescence change of 0.04 μM IF3_DL_ (sky blue) or IF3_Alx488_ (no acceptor control, grey) during binding to 0.12 μM 30S. Black lines indicate non-linear fitting with two exponential terms (Equation 1). Volts in the y-axis denotes the fluorescence change measured by the instrument photomultiplier. (**B**) *k_app1_* and (**C**) *k_app2_* dependence as a function of 30S concentration upon formation of the 30S-IF3 complex. Continuous lines in B and C show the non-linear fitting with hyperbolic function. Error bars represent standard deviations resulting from the independent fitting of 5–7 replicates for each 30S concentration.

Binding of IF3_DL_ to the 30S ribosomal subunit resulted in a biphasic increase of fluorescence over time with both apparent rates saturating with 30S concentration (Figure 2, Supplementary Figure S5A). The overall opening of the factor occurs in two steps with amplitudes of 1.4 ± 0.2 V and 0.9 ± 0.1 for the first and second reactions, respectively (Supplementary Figure S6A). Kinetic analysis of each apparent rate dependency of the 30S concentration indicated that after the initial binding (10^3^ μM^–1^ s^–1^, (4)), IF3 stretches at a velocity of 113 ± 8 s^–1^ (Figure 2B, Table 1). Subsequently, IF3 further extends at a saturating apparent velocity of 5.2 ± 0.2 s^–1^ (Figure 2C, Table 1). This data is in agreement with time resolved chemical probing and NMR spectroscopy analysis showing that IF3C and IF3N sequentially interact with the 30S ribosome (16,38). Thus, our results show that IF3_DL_ rapidly transits from a more compact state in solution to an extended state when bound to 30S (Supplementary Figure S6). To further explore the conformation of the factor in solution, we used Molecular Dynamics of free IF3 starting from three different states of the factor. In all cases, the distance between the domains, as measured from the labelled cysteines, is reduced compared to the starting condition, indicating a compaction of IF3 to an average of 33 Å. Furthermore, a comparison between *wt* IF3, the E166C mutant, and labelled IF3 confirms that the overall tendency for the factor is to reduce the interdomain distance in solution (Supplementary Figure S6C–E).

**Table 1.** Saturating velocities of IF3_DL_ conformational changes

Complex	Ligand	*k*_app1_ (s^–1^)*	*k*_app2_ (s^–1^)*	*k_AV_* (s^–1^)**
30S	IF3_DL_	113 ± 8	5.2 ± 0.2	64 ± 4
30S-IF3	IF2	11 ± 1	-	-
30S-IF_3/2_	IF1	3 ± 1	0.47 ± 0.02	1.3 ± 0.2
30S pre-IC (w/mRNA)	fmet-tRNA	***	4 ± 1	-
30S pre-IC (w/fMet-tRNA)	mRNA	0.22 ± 0.01	-	-
30S IC	50S	12.9 ± 0.4	0.54 ± 0.01	5.5 ± 0.2

*V_max_ calculated with Equation (2).

**V_max_ of the averaged rates obtained with Equation (3). Standard deviations were calculated according with the error propagation rules for sum and product (Equation 4)

***IF3 conformational change upon fMet-tRNA^fMet^ binding was best described by a linear dependence on concentration of 17 μM^–1^s^–1^

To position the factor on the 30S-IF3_DL_ complex, we compared our FRET efficiencies to all combinations of IF3C and IF3N sites retrieved from available structures. The structural modelling of IF3_DL_ combined with FRET changes suggest that in the absence of any other ligand, IF3 acquires the most extended layout with IF3C likely occupying the C1 site and IF3N moving into the N_R_ site with an interdomain distance of about 60 Å (Supplementary Table S1). This IF3 layout is affected by all ligands of the 30S subunit, with IF1 and IF2 bringing IF3 domains closer (See below). IF3 was shown to readily dissociate from the 30S in the absence of other factors (4,7), indicating that IF3C positioning at the C1 and IF3N at the N_R_ binding sites constitute a kinetically unstable configuration of the factor.

### IF1 and IF2 induce an IF3 conformation suitable for initiator fMet-tRNA^fMet^ binding

**Figure 3.**
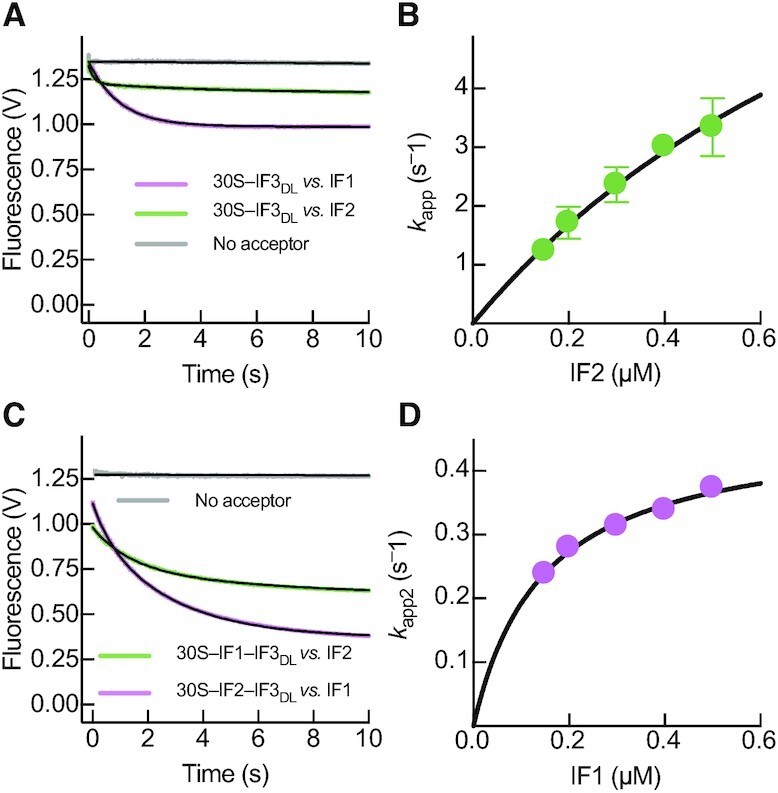
IF1 and IF2 recruitment to the 30S subunit bring IF3 domains in proximity. Stopped-flow analysis of IF3_DL_ conformational transitions during binding to the 30S subunit and binding of IF1 and IF2 to the 30S–IF3_DL_ complex. (**A**) Time courses of 0.5 μM IF1 (pink) or IF2 (green) binding to 0.05 μM 30S–IF3_DL_ complex. (**B**) Concentration dependence of the apparent rates of IF3_DL_ closure upon binding of IF2 to the 30S-IF3_DL_ complex. (**C**) Time traces of IF3_DL_ fluorescence changes as function of 0.5 μM IF1 (pink) or 0.5 μM IF2 (green) binding to 0.05 μM 30S–IF3_DL_–IF2 or 30S–IF3_DL_–IF1 complexes, respectively. (**D**) Concentration dependence of *k*_app2_ of the main IF3_DL_ closure reaction upon binding of IF1 to the 30S–IF3_DL_–IF2 complex. Grey time courses in (A) and (C) represent reactions with IF3_Alx488_ (No acceptor control) to verify that the fluorescence signal produced by IF3_DL_ depended on distance changes between fluorophores. Continuous lines represent non-linear fitting with exponential (A and C, Equation 1) or hyperbolic (B and D, Equation 2) functions. Each time trace results from the average of 5–7 independent measurements. Volts in the y-axis of (A) and (C) denotes the fluorescence change measured by the instrument photomultiplier. Error bars in (B) and (D) represent standard deviations from the independent analysis of each replicate.

A previous report suggested IF2 is recruited shortly after IF3 to build the 30S–IF_2,3_ intermediate complex (4). Subsequently, IF1 joins and triggers an overall kinetic stabilization of the resulting 30S–IFs complex. Binding of IF2 and IF1 to 30S–IF3_DL_ complex promoted a reduction of the distance between IF3 domains as observed from the decrease of IF3_DL_ fluorescence, in agreement with single molecule analysis (21). However, IF1 and IF2 showed different apparent kinetics (Figure 3A). IF2-dependent closure of the 30S-bound IF3_DL_ occurred at a single step and saturated at 11 ± 1 s^–1^ (Figure 3B, Table 1), indicating that IF3 rearranges 20 times slower than the initial bimolecular reaction (4). The kinetics of IF3_DL_ accommodation during IF2 binding were also influenced by the presence of IF1, slowing the reaction (Figure 3C). IF1-dependent compaction of IF3_DL_ occurs in two phases with the second reaction accounting for 60% of the total amplitude and saturating at *k_app2_*= 0.47 ± 0.02 s^–1^ (Figure 3D, Table 1, Supplementary Figure S7A). The kinetics of the main IF1-dependent distance reduction of IF3_DL_ were 24-fold slower than those observed for IF2 (Figure 3D) and with a lower amplitude dependence on factor concentration (Supplementary Figure S5B, C). The main closure of IF3_DL_, triggered by IF1, was preceded by a faster reaction (*k*_app1_ = 3 ± 1 s^–1^), yet accounting for a smaller amplitude (40%, Supplementary Figure S7A,B). The overall speed of IF1-dependent compaction of IF3_DL_ in the 30S–IFs complex occurred at an average rate of 1.3 ± 0.2 s^–1^ (Table 1, Supplementary Figure S7C).

Structural modelling of IF3_DL_ on the 30S subunit suggests that IF1 may enhance the movement of IF3C from the C1 to the C2 binding site (Figure 1), partially accounting for the observed reduction of interdomain distances (Supplementary Figures S3 and S4). Additionally, the extent of FRET reduction in combination with the structural modelling suggests that IF3N could be concomitantly displaced towards IF3C, a shift cooperatively maximized by the presence of both IF1 and IF2 (Figure 3C). The structural analysis suggested a 10 Å distance reduction between domains and up to 20 Å between the two fluorophores (Supplementary Table S1). Altogether, our data indicate that the intermediate 30S–IFs complex promotes an IF3 layout where IF3C moves towards C2, near the P site, and IF3N may acquire a rather more dynamic state, dissociating from the N_R_ site and fluctuating towards the P site along the N_T_ intermediates. While occupation of the C2 binding site would interfere with the formation of inter-subunit bridges (reviewed in (39)), positioning of IF3N would contribute to shape the fMet-tRNA^fMet^ binding site. The 30S–IFs complex assembles in ≈30 ms (4) and rearranges in ≈ 1 s (this work). This intermediate state of translation initiation is characterized by a high affinity of IF3 for the 30S, enhancing the fidelity of the process by preventing the premature binding of the 50S subunit (5,9), and by promoting decoding of the mRNA start site (10) (Figure 1A).

### IF3N senses fMet-tRNA^fMet^ recruitment by IF2

**Figure 4.**
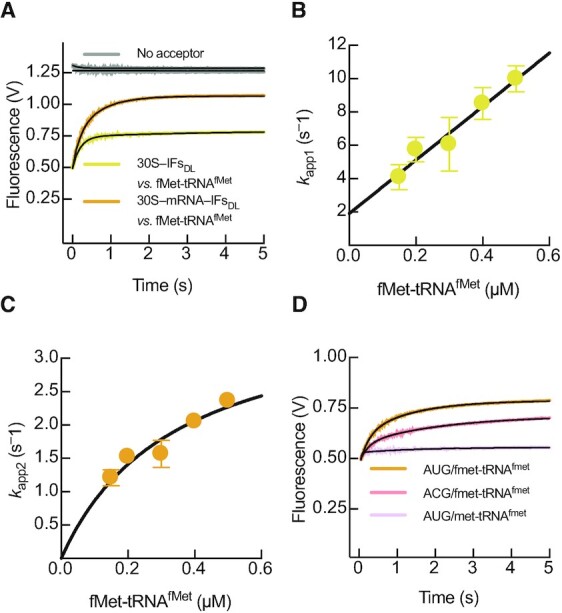
fMet-tRNA^fMet^ and decoding of the mRNA displace IF3 domains apart from each other. (**A**) Time courses of IF3_DL_ fluorescence changes during binding of 0.5 μM fMet-tRNA^fMet^ to 0.05 μM 30S pre-IC in the presence (orange) or absence (yellow) of the mRNA. fMet-tRNA^fMet^ concentration dependence of the fast (**B**) and slow (**C**) apparent rates of IF3_DL_ opening upon binding of the initiator tRNA to 30S pre-ICs. Grey time courses in (A) represent reactions with IF3_Alx488_ (No acceptor control). (**D**) Time courses of IF3_DL_ opening upon binding of 0.15 μM fMet-tRNA^fMet^ to 30S complexes lacking an initiation codon (pink) or binding of non formylated Met-tRNA^fMet^ in the presence of a canonical AUG start codon (light violet). Continuous lines represent non-linear fitting with exponential (A and D, Equation 1), linear (B) or hyperbolic (C, Equation 2) functions. Each time trace in (A) and (D) results from the average of 5–7 independent measurements. Volts in the y-axis of (A) and (D) denotes the fluorescence change measured by the instrument photomultiplier. Error bars in (B) and (C) represent standard deviations from the independent analysis of each replicate.

When a canonical start codon of the mRNA is present in the P site, the initial interaction of fMet-tRNA^fMet^ with the 30S complex is followed by codon–anticodon formation. The resulting 30S IC, in comparison to the pre-IC, displays an increased dissociation rate constant (*k*_off_) of IF3 from the 30S subunit (4,5). To probe how IF3 interdomain dynamics senses initiator tRNA recruitment, we first measured fMet-tRNA^fMet^ binding to 30S complexes containing IFs but lacking the mRNA (Figure 4A). The resulting fluorescence traces increased with time in a single phase, indicating a single step re-accommodation of either domains of IF3_DL_, moving away from each other (Figure 4A). Kinetic analysis obtained at increasing concentrations of fMet-tRNA^fMet^ showed a linear dependence of velocities with tRNA concentrations, consistent with IF3_DL_ monitoring the initial bimolecular interaction of the fMet-tRNA^fMet^ with 30S pre-IC lacking the mRNA (Figure 4B). Fitting the *k_app_* dependence on tRNA concentration with single step interaction model using linear regression indicates that IF3 opens at *k*_1_ = 17 μM^–1^ s^–1^ and can close at *k*_–1_ = 1 s^–1^. Similar kinetics were observed by using tRNA and IF3 as FRET pairs (37), indicating that IF3_DL_ directly monitors the initial fMet-tRNA^fMet^ binding.

Thus, the initial interaction of fMet-tRNA^fMet^ entails the partial opening of IF3, probably as a consequence of transiently displacing IF3N towards the N_R_ site to a position similar to that observed in cryo-EM reconstructions of the 30S pre-IC (22). Indeed, the structural modelling suggests a 10 Å distance increase between the fluorescent dyes, with IF3N likely occupying the N_T I-III_ states (Supplementary Table S1). Altogether, the formation of the 30S pre-IC lacking the mRNA entails an IF3 layout where IF3C would be positioned at the C2 site and IF3N is transiently pushed by the initiator tRNA towards the N_R_ site, yet interacting with the tRNA elbow along N_T_ states. The reaction is rapid under our experimental conditions (17 ± 1 s^–1^ at 1 μM of fMet-tRNA^fMet^) and IF3 accommodation during 30S pre-IC formation shall take around 50 ms.

### The displacement rate of IF3C limits 30S IC formation

The kinetics of the IF3_DL_ FRET signal as a function of fMet-tRNA^fMet^ recruitment depended on the presence of the mRNA. In contrast to the absence of mRNA, mRNA-programmed 30S complex appeared biphasic in time, indicating a further accommodation of IF3_DL_ following the initial binding of fMet-tRNA^fMet^ (Figure 4A). The second phase was largely affected by the nature of the initiation codon (Figure 4D). Furthermore, IF3_DL_ layout did not change when an initiator tRNA lacking the formyl group was mixed to 30S pre-IC complexes (Figure 4D). Thus, IF3 re-positioning in the 30S IC requires a formylated initiator tRNA and a canonical initiation codon. Kinetic analysis of time courses obtained at increasing concentrations of fMet-tRNA^fMet^ showed that the velocities of the IF3_DL_ accommodation step in 30S pre-ICs containing the mRNA saturate at 4 ± 1 s^–1^ (Figure 4C, Table 1, Supplementary Figure S5D). These experimental data, coupled with the structural modelling, support a model where IF3 accommodates the fMet-tRNA^fMet^ on the P site through movements of IF3N (Figure 1) combined with IF3C, likely moving from the C2 position towards C1; altogether, the IF3 domains move away from each other. However, the complexity of the above reactions precluded us to obtain accurate information on the kinetics of mRNA start site decoding.

**Figure 5.**
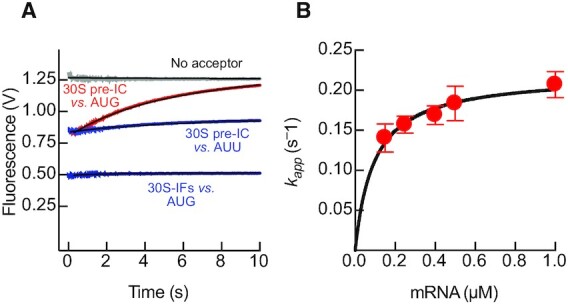
Decoding of the mRNA start site rate limits translation initiation. (**A**) Time courses of 0.5 μM mRNA binding to 0.05 μM 30S pre-IC containing IF3_DL_ in the presence (red) or absence (light blue) of fMet-tRNA^fMet^. Binding of mRNA coding the AUU start site is shown in blue. Grey trace represents the reaction using IF3_Alx488_ (No acceptor control). Volts in the y-axis denotes the fluorescence change measured by the instrument photomultiplier. (**B**) mRNA concentration dependence of the apparent rates of IF3_DL_ closure upon binding to the 30S pre-ICs containing fMet-tRNA^fMet^. Continuous lines represent non-linear fitting with exponential (A, Equation 1) or hyperbolic (B, Equation 3) functions. Each time trace in (A) results from the average of 5–7 independent measurements. Error bars in (B) represent standard deviations from the independent analysis of each replicate.

To overcome this limitation, we set up an experiment where 30S pre-ICs were pre-bound to fMet-tRNA^fMet^ and mixed with increasing concentrations of mRNA. Mixing of the mRNA with 30S pre-ICs resulted in a single step increase of IF3_DL_ fluorescence, indicating an increase of distance between IF3 domains with velocities saturating at 0.22 ± 0.01 s^–1^ (Figure 5A, B, Table 1, Supplementary Figure S5E). The increase of fluorescence observed in this experimental setup is likely the result of the displacement of IF3C from C2 towards the C1 binding site (Figure 1) rather than repositioning IF3N, which appears mostly affected by initiator tRNA (see above). Accordingly, structural modelling of IF3_DL_ on 30S IC structures indicate that the IF3C displacement accounts for 10 Å change of distance between the fluorophores on IF3 domains (Supplementary Figures S3 and S4). A comparison of the velocities for all IF3 movements suggests that during decoding of the mRNA start site, the displacement of IF3C towards the C1 binding site is the slowest. Altogether, our results show that the IF3C displacement is the slowest step, rate-limiting the progression of 30S IC formation and taking about 3 s.

### AUG decoding unlocks IF3C and 70S IC formation

**Figure 6.**
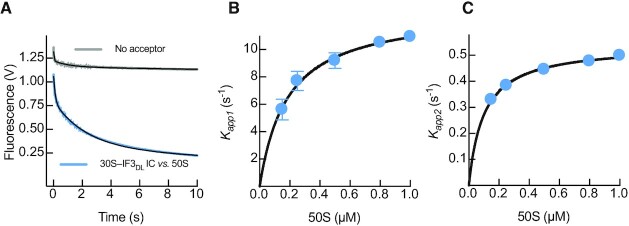
70S IC formation is accompanied by IF3 compaction prior to dissociation. (**A**) Time traces of IF3_DL_ fluorescence change upon binding of 0.5 μM 50S to 0.05 μM 30S IC (sky blue). Grey trace indicates a control using IF3_Alx488_ (No acceptor control). Volts in the y-axis denotes the fluorescence change measured by the instrument photomultiplier. (**B**) 50S concentration dependence of the initial compaction rates (*k*_app1_) of IF3_DL_. (**C**) 50S concentration dependence of the subsequent closure apparent rates (*k*_app2_) of IF3_DL_. Continuous lines represent the non-linear fitting with exponential (A, Equation 2) or hyperbolic (B, Equation 3) functions. Each time trace results from the average of 5–7 independent measurements. Error bars represent standard deviations from the independent analysis of each replicate.

Joining of the 50S to 30S ICs results in an unstable 70S pre-IC, which upon release of IFs allows the formation of a 70S IC capable of translating the in-frame mRNA (5,9,12). The reaction appears to be sequential (12) and to be tightly regulated by IF3 (5,6,9,40). 50S joining to 30S ICs with IF3_DL_ resulted in a fluorescence decrease over time, indicating a closure of the factor during dissociation (Figure 6A). The reaction was characterized by an initial fast compaction of IF3 that accounted for 36% of the total amplitude and saturated at *k*_app__1_ = 12.9 ± 0.4 s^–1^ (Figure 6B, Table 1, Supplementary Figure S8A). The initial compaction of IF3_DL_ was followed by a further closing of the factor with slower apparent rates and saturating at *k*_app__2_ = 0.54 ± 0.01 s^–1^ (Figure 6C, Table 1). Altogether the mechanism of IF3 compaction in the 70S pre-IC and subsequent release occurs at an average rate of 5.5 ± 0.2 s^–1^, very similar to previous reports that monitored IF3 dissociation using fMet-tRNA^fMet^ as FRET pair (5,12) (Supplementary Figure S8B).

**Figure 7.**
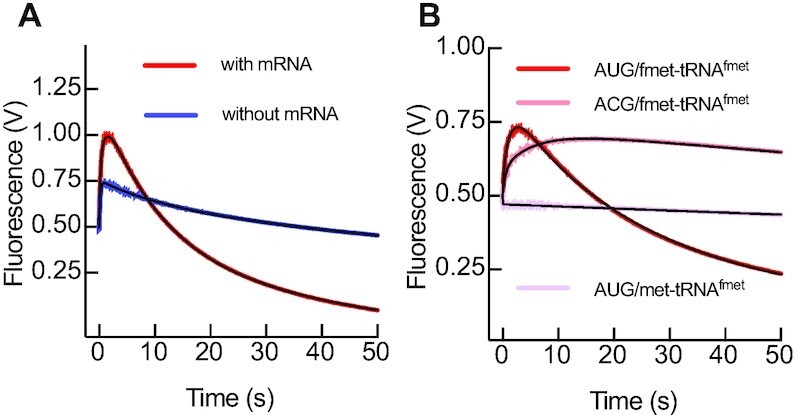
IF3 dynamics during the kinetic checkpoint of 70S IC formation. (**A**) Time courses of IF3_DL_ fluorescence change during the concomitant binding of 0.5 μM fMet-tRNA^fMet^ and 50S to 0.05 μM 30S pre-IC in the presence (red) or absence (blue) of the mRNA. (**B**) Time courses using the same set-up described in (A) to assess IF3 movements in complexes lacking an initiation codon (pink) or the formyl group in the initiator tRNA (violet). Continuous lines represent the fitting with exponential functions (Equation 2). Volts in the y-axis denotes the fluorescence change measured by the instrument photomultiplier. Each time trace results from the average of 5–7 independent measurements.

When we measured the concomitant arrival of fMet-tRNA^fMet^ and 50S to 30S pre-ICs with and without mRNA, we could observe a time dependence of IF3_DL_ fluorescence change indicating the opening of the factor due to initiator tRNA binding (increase from 0.5 to 1.0) followed by the closure of IF3 caused by the ejection of the factor from the resulting 70S IC (decrease from 1.0 to 0.0) (Figure 7A). The rate of the latter was very similar to that monitored for mRNA binding to 30S pre-ICs that contained initiator tRNA (Figure 5). In the absence of the mRNA, IF3_DL_ in the 30S pre-IC still sensed the arrival of fMet-tRNA^fMet^; however, IF3 compaction or dissociation was missing, and the factor appeared to adopt its initial layout (Figure 7A). Consistently, when 30S pre-ICs were programmed with an mRNA lacking an initiation codon, IF3 responded to fMet-tRNA^fMet^ binding but failed to dissociate (Figure 7B). In the absence of the formyl moiety of the initiator tRNA, IF3_DL_ did not show any conformational change, indicating that the IF2 mediated recruitment of fMet-tRNA^fMet^ strongly influence the reaction (Figure 7B) in agreement with the observed response of IF3_DL_ to Met-tRNA^fMet^ in the 30S IC (Figure 4D). Altogether, the kinetic checkpoint during late events of translation initiation is orchestrated by a series of movements of IF3 due to the initiator tRNA arrival, start codon selection and dissociation of the factor from the ribosome.

## DISCUSSION

**Figure 8.**
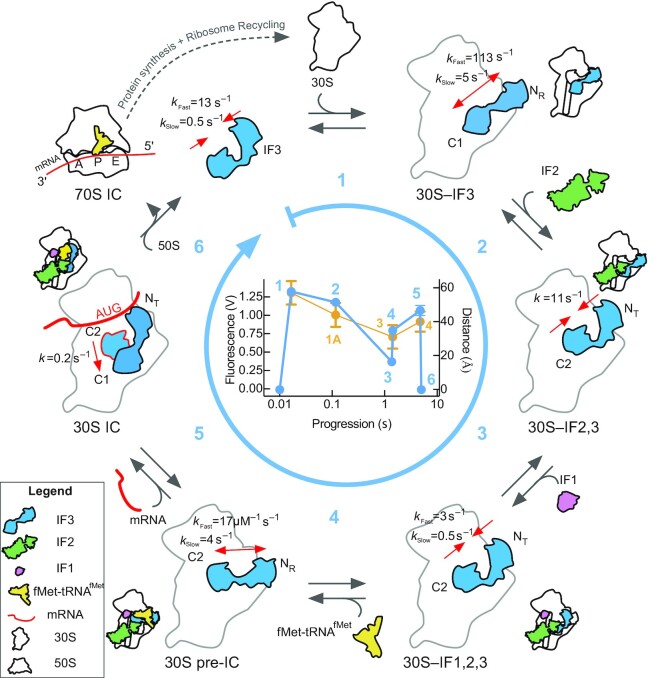
Scheme of IF3 motions during bacterial translation initiation. Conformational accommodations of IF3 along the full cycle of translation initiation was monitored by intramolecular FRET between dyes specifically linked to each domain of the factor and structural modelling of IF3_DL_ on available 30S IC structures (22). The round arrow (sky blue) indicates the direction of complex progression by measuring IF3_DL_ accommodation as a function of the 30S subunit (white), IF2 (green), IF1 (pink), fMet-tRNA^fMet^ (yellow), the mRNA (red), and the 50S subunit. Sky blue numbers indicate the main reactions studied here. Red arrows indicate the directionality and average rates of IF3 movements measured here. Alternative binding sites for IF3N are: N_R_ (30S-bound) and N_T_ (fMet-tRNA^fMet^- bound). Alternative binding sites for IF3C are indicated as C1 or C2. 30S pre-IC indicates the pre-initiation complex while mRNA is indicated by a red line in the 30S and 70S ICs. Inset: Comparison between the amplitude changes of IF3_DL_ for all the reactions measured here (sky blue line, left axis) and distances between the fluorophores on the corresponding complexes as analysed by molecular modelling (orange, right axis). Orange numbering refer to the 30S PICs 1A, 3 and 4 complexes from (22). *x*-axis represent the progression time of each reaction as calculated from the kinetic analysis. Error bars represent standard deviations from the total amplitudes for each reaction (sky blue) or from the distance distribution of IF3_DL_ fluorophores calculated by structural modelling (orange, Supplementary Figures S3 and S4, Table S1).

IF3 enhances the fidelity of translation initiation with three defined functions. IF3 prevents the premature joining of the large 50S subunit by impairing inter-subunit bridges B2a and B2b (reviewed in (39)). The factor increases the rate of the P site codon-anticodon interaction between fMet-tRNA^fMet^ and the initiation triplet of the mRNA (10). In late complexes, IF3 orchestrates a kinetic checkpoint of the ribosome entering the elongation phase of protein synthesis (41). This work expands the chronological framework of events that were proposed solely by structural analysis (22), assigning the positions on the ribosome and the kinetics of both IF3 domains in all translation initiation intermediate complexes. In addition, the structural and kinetic landscape were tested with non-initiator tRNAs and mRNAs with non-canonical initiation triplets, providing a comprehensive model depicting how the factor performs its fidelity function. Our FRET approach, in combination with real-time measurements and molecular modelling of available 30S IC structures, describe the dynamics of IF3 interdomain transitions along all functional intermediates during 70S IC formation: from binding to dissociation and recycling of the factor (Figure 8).

The anti-association activity of IF3 was shown to be maximised by the cooperative action of IF1 and IF2 (5). Our results indicate that both factors enhance the movement of IF3C towards the C2 binding site, near the P site, blocking inter-subunit bridge B2a (Figures 3 and 8). Similar analysis by single molecule assays showed that both IF1 and IF2, can independently promote the movement of IF3 domains closer to each other (21). However, the concomitant binding of both factors produced a less pronounced effect (21). In our experimental model and approach (Inset in Figure 8), the interdomain distance reduction induced by IF1 and IF2 suggests a movement of IF3N towards IF3C, as compared to the structural analysis of available 30S complexes obtained by cryo-EM (22). This is an appealing model, as it would entail a progressive induced fit for the fMet-tRNA^fMet^ binding site architecture. In this context, IF3C moving to the C2 site would be coupled to the dissociation of IF3N from the N_R_ site to adopt a dynamic layout that is ready to interact with the initiator tRNA (Step 3 in Figure 8).

The induced fit model for initiator tRNA binding would also promote and enhance the kinetics of start codon recognition on the mRNA through two interactions. First, IF3C in the C2 site is positioned near the P site in 30S–IFs complexes, providing interaction points with initiator tRNA near the codon anticodon duplex (22). Second, the IF3N displacement from the N_R_ binding site towards the P site would allow the domain to interact with the initiator tRNA elbow (42). Thus, formation of the 30S–IFs complex allows IF3 to acquire a conformation capable of stimulating the binding of initiator tRNA and favouring start codon selection. Indeed, when the tRNA binding architecture is altered by either using mutant tRNAs or by shortening the linker connecting IF3C to IF3N the outcome is an incorrect decoding of the mRNA start site and fitness loss *in vivo* (42,43). Our results show that the binding of initiator tRNA increases the interdomain distance in IF3 with kinetics corresponding to a bimolecular interaction followed by a conformational change (Figure 3A-C). The initial opening of IF3 can be interpreted as fMet-tRNA^fMet^ transiently displacing IF3N towards the E site, occupying the N_T_ binding sites (Step 4 in Figure 8). The transient displacement of IF3N appears to be mRNA or start codon independent (Figure 4A and D) and dependent on the formyl group of the initiator tRNA (Figure 4D). Thus, IF3 senses the arrival of tRNAs where IF2 (C2 domain) preferentially selects the formylated Met-tRNA^fMet^ (44). In this context, the following rearrangements of IF3N may enhance the kinetics of the mRNA start codon selection as they are absent in 30S complexes lacking the mRNA or an initiation codon (Figure 7). Indeed, cryo-EM reconstructions of early 30S pre-ICs showed that fMet-tRNA^fMet^ lays over IF3N at the 30S platform near the E site and should move towards the P site for 30S IC formation (22).

Our results show that during fMet-tRNA^fMet^ binding to mRNA-programmed 30S pre-ICs, the transient movement of IF3N towards the N_R_ site was followed by a slower reaction promoting a further opening of IF3 (Figure 4A). Our structural modelling suggests that the further opening is compatible with IF3C being displaced away from the C2 towards the C1 site (Supplementary Figure S4, Table S1 and Figure 4A). The latter displacement of IF3C was strictly dependent on the mRNA and initiation codon, emphasizing that IF3C at the C2 site is the main contributor to enhancing start codon selection (10) (Figure 5A and Step 5 in Figure 8). Altogether, the kinetics of start codon selection are enhanced by IF3C positioned in the C2 site and by IF3N in the N_T_ conformations (Step 5 in Figure 8). Importantly, our data show that IF3C moving towards the C1 site during start codon selection rate limits translation initiation and unlocks 70S IC formation (Figure 5). Contrary to our findings, single molecule measurements reported that 30S IC formation entailed the compaction of IF3, albeit largely agreeing on an extended state of the factor in the 30S pre-IC complex lacking fMet-tRNA^fMet^ (21). There are differences in the experimental setup, factor engineering, labelling positions, and fluorophores used that could explain the discrepancies. Furthermore, each experimental approach, bulk and SM, possess distinct limitations. Bulk measurements, for instance, record the overall and averaged FRET states, whereas SM has the advantage to dissect the individual contribution of each population present in the dynamic system. Nevertheless, the SM approach is mRNA dependent as it is used to tether the 30S to the observation plane. Thus, the mRNA contribution to IF3 dynamics could not be assessed directly and precluded measurements of fMet-tRNA^fMet^ binding to 30S pre-IC lacking the mRNA. Particularly, the FRET pair used in the Elvekrog and Gonzalez study showed very low FRET for complexes lacking the tRNA, which may have hindered a further transient opening of IF3 during tRNA binding and decoding of the initiation codon (21). Nevertheless, they also observe a defined signal indicating the compaction of IF3 only for complexes carrying an AUG and fMet-tRNA^fMet^. Our study does not observe a similar compaction response prior to 50S joining and during IF3 dissociation (Figure 6).

The rates of 70S IC formation and of IF3 dissociation depend on the mRNA bound to the ribosome, entailing a kinetic checkpoint during late steps of translation initiation (5). Our results show how the inter-domain dynamics of IF3 participate during late events of translation initiation. Binding of the 50S subunit to canonical 30S ICs resulted in a rapid reduction of the interdomain distances of IF3 followed by a slower step of further interdomain distance reduction. Thus, 50S binding to the 30S IC entails a rapid compaction of IF3 before release which may resemble the factor state assigned to the 30S IC in previous studies (21,22). Previous rapid kinetics measurements of subunit joining used experimental conditions similar to those in the SM study and concluded that IF3 dissociates prior to 50S arrival (40). This has been largely disproved by cryo-EM, SM, and rapid kinetics reports (5–7,21–23). Thus, the compact IF3 layout observed in the 30S IC by the SM study may resemble the factor in a ready to go conformation. In our study a compacted conformation of IF3 arises in the 70S pre-IC, after the 50S initially interacts with the 30S IC (Figure 6A,B).

The initial rapid closure of IF3 was followed by a slower rearrangement bringing IF3 domains in closer proximity, reaching a conformation similar to that of free IF3 in solution (Figure 6A, C and step 6 and inset in Figure 8). The average rates of IF3 closure during 70S IC formation were found similar to those described using the tRNA as FRET donor to IF3 (5 s^–1^, Figure 6B) (5,12). Thus, the overall IF3 compaction during 70S IC formation shows that the dissociation of the factor occurs through two defined steps (Figure 6A). The full dynamic range of IF3 functioning during the late steps of translation initiation was observed when 30S pre-ICs were mixed with fMet-tRNA^fMet^ and 50S in a single reaction (Figure 7A). In this experimental setup, IF3 sequentially moved its domains to accommodate initiator tRNA (IF3N) and to allow 50S joining (IF3C) (Figure 7A). The latter reaction limited the overall rate of IF3 dissociation, indicating that mRNA start site recognition is rapid. The lack of mRNA on the 30S pre-IC abolished IF3C displacement and dissociation; however, IF3N moved towards the N_TI-III_ positions (Figure 7A, Supplementary Figure S4). Thus, IF3 can kinetically control the progression towards elongation by rapidly accommodating its domains to enhance the canonical codon anticodon interaction, either by a direct sensing from IF3C or through allosteric modulation of the P site together by a dynamic positioning of IF3N near the initiator tRNA. Indeed, replacing the initiation codon with a non-initiation codon resulted in negligible transitions towards 70S ICs (Figure 7B).

In a cellular context, the progression of translation initiation would result in at least two long-lasting intermediate complexes, 30S–IFs and the 30S pre-IC (containing the mRNA and initiator tRNA but lacking a codon-anticodon duplex). The former would ensemble in less than 30 ms and rearrange in around 1 s, being able to reject the 50S by maximal occupancy of the C2 site by IF3C (Figure 1B), and IF2, through conformational signalling (45). On the other hand, the 30S–IFs complex can rapidly recruit the initiator tRNA at the P site by providing a functional binding pocket with IF3C in the C2 site, IF3N ready to interact with the tRNA, and IF2 interacting with the formyl group of the initiator tRNA. The transition towards a 30S pre-IC, harbouring all ligands, would be limited by the availability of mRNA translation initiation regions (TIRs). Finally, upon decoding of the mRNA start site, the displacement of IF3C from the C2 site induces 30S IC locking, a reaction that rate limits the overall progression rate of translation initiation (Figure 8). This step would allow to build 70S pre-ICs in less than 250 ms followed by a rapid and directional cascade of reactions that ultimately leads to a productive and ready-to-elongate 70S IC (12).

In summary, we describe the functional conformations of IF3 defining with high accuracy the temporal framework covered by both domains of the factor in correlation with all events taking place during the translation initiation process. Altogether, this work allows to accurately assign the speed and nature of elemental movements of IF3 behind its essential fidelity function.

## Supplementary Material

gkab522_Supplemental_Files
