# Supplementary material for: The dynamic cycle of bacterial translation initiation factor IF3
Source: Nucleic Acids Res. 2021 Jun 23;49(12):6958–70. doi: 10.1093/nar/gkab522 (PMC8266586; doi:10.1093/nar/gkab522)
Supplement: gkab522_Supplemental_Files [file gkab522_supplemental_files.zip › Supplementary_Data_Nakamoto_et_al_corrected.pdf]

Supplementary Information for:

The dynamic cycle of bacterial translation initiation factor IF3

Jose A. Nakamoto[1], Wilfredo Evangelista[1], Daria S. Vinogradova[2,3], Andrey L. Konevega[2,4,5], Roberto Spurio[6], Attilio Fabbretti[6], and Pohl Milón[1,*]

[1] Laboratory of Applied Biophysics and Biochemistry, Centre for Research and Innovation, Health Sciences Faculty, Universidad Peruana de Ciencias Aplicadas (UPC), Lima 15023, Peru

[2] Petersburg Nuclear Physics Institute, NRC "Kurchatov Institute", Gatchina 188300, Russia

[3] NanoTemper Technologies Rus, Saint Petersburg 191167, Russia

[4] NRC "Kurchatov Institute", Moscow 123182, Russia

[5] Peter the Great St. Petersburg Polytechnic University, Saint Petersburg 195251, Russia

[6] Laboratory of Genetics, School of Biosciences and Veterinary Medicine, University of Camerino, Camerino 62032, Italy

* To whom correspondence should be addressed. Tel: +51 1 3133333 (2762); Fax: +51 1 3133334; Email: pmilon@upc.edu.pe

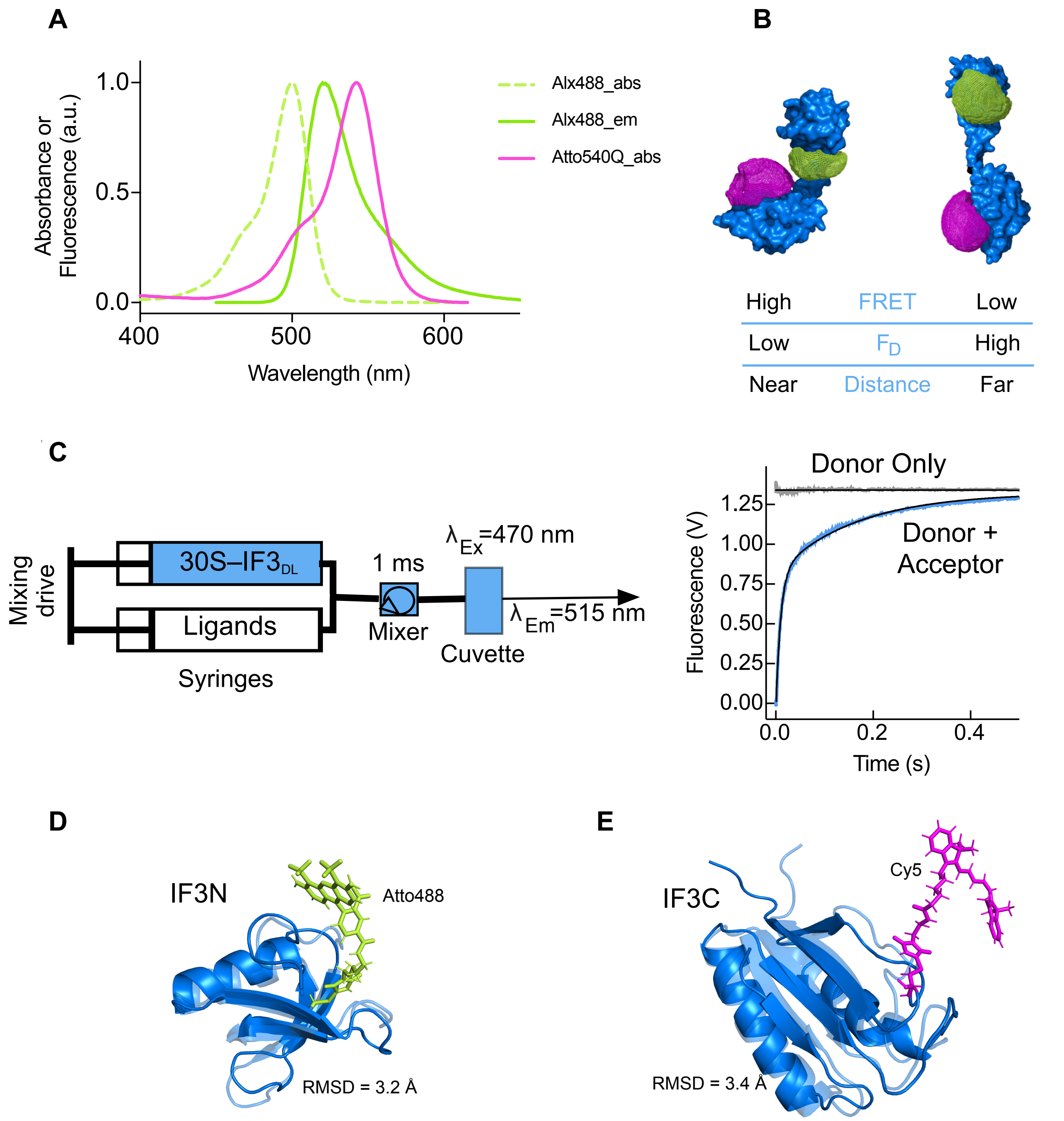

Supplementary Figure 1. Experimental approach. $IF3_{DL}$ for dynamic measurements of the factor on the 30S platform. (A) Absorption and emission spectra of Alexa488 fluorescent dye (green) and Atto540Q quencher (magenta). (B) Schematic example of $IF3_{DL}$ arrangements. IF3 surface is shown in blue while meshed volumes indicate the spatial volume that dyes occupy (colors are as (A)). (C) Scheme of Stopped-Flow experimental set-up and the typical signal read out upon mixing 30S–$IF3_{DL}$ with a 30S binder (sky blue trace). In order to assign the signal as FRET, the same experiment is performed in the absence of the acceptor, $IF3_{N\ Alx488}$ (grey trace). Comparisons of the

labelled IF3N (D) and IF3C (E) as obtained by Molecular Dynamics (blue) with the *wild type* structure (light blue). RMSD stands for Root Mean Square Deviation in Armstrong.

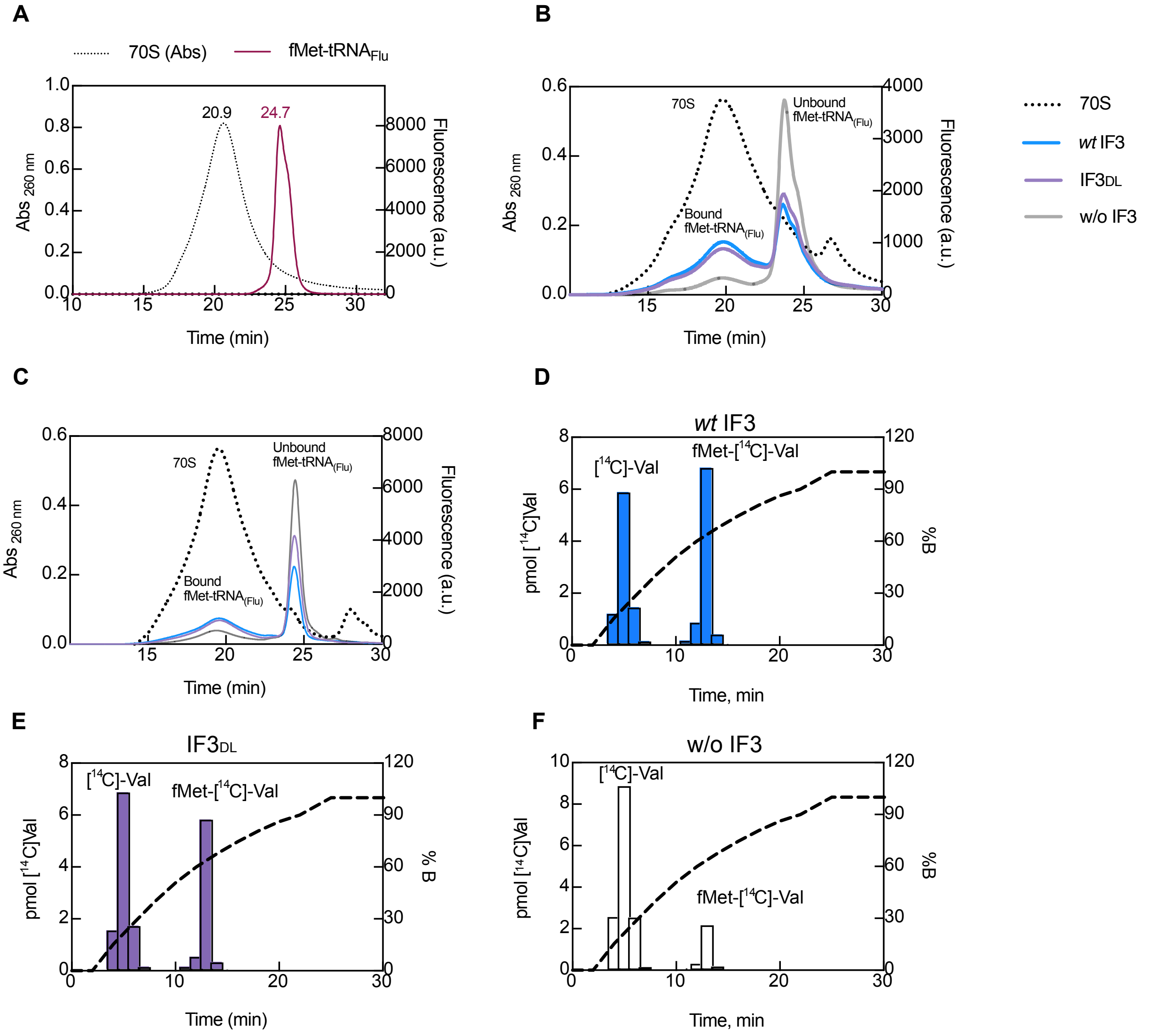

Supplementary Figure 2 (Related to Figure 1). $IF3_{DL}$ activity assessment. (A) HPLC chromatogram of individual runs for 0.1 µM 70S ribosomes (dotted trace, Abs 260n m, left axis) and 0.2 µM $fMet\text{-}tRNA^{fMet}$ (Flu) (red continuous trace, Ex=460 nm, Em=540 nm, right axis). SEC BioSuite 450HR was used under isocratic flow (0.5 mL/min) in $TAKM_7$ buffer. 70S ribosomes and $fMet\text{-}tRNA^{fMet}$ (Flu) eluted with retention times of 20.9 min and 24.7 min, respectively. (B) Comparison of HPLC chromatograms for 70S ICs formed in multiple turn over conditions for *wt* IF3 (blue), $IF3_{DL}$ (purple) or without the factor (grey) as measured by fluorescence of $fMet\text{-}tRNA^{fMet}$ (Flu) (right axis). 70S ICs were incubated for 30 min at 37˚C using 0.1 µM 70S tight coupled ribosomes, 0.01 µM IF3s, 0.3 µM IF1 and IF2, 0.2 µM fMet-tRNAfMet (Flu), 0.3 µM mRNA mMVF and 0.1 mM GTP in $TAKM_7$ buffer. Dotted trace corresponds the absorbance at 260 nm (left

axis). (C) Comparison of HPLC chromatograms (as in panel B) for 70S ICs formed under stoichiometric conditions for IF3 (0.1 µM) and 70S ribosomes (0.1 µM). (D-F) HPLC chromatograms for dipeptide analysis using a C8 column eluted with an acetonitrile gradient (0-65 % + 0.1 % TFA). Fractions (1 mL) were collected and the [^{14}C]-Val radioactivity was measured using scintillation counting. 70S ICs (0.1 µM) were formed as in (A) with *wt* IF3 (D), IF3DL (E), or without IF3 (F) and mixed with 0.2 µM EF-Tu-GTP-[^{14}C]Val-tRNA^{Val} . Further details are provided in materials and methods section. Dotted lines show the acetonitrile gradient (right axis). The beta-counter measured each fraction until the standard error was bellow 5% of the mean.

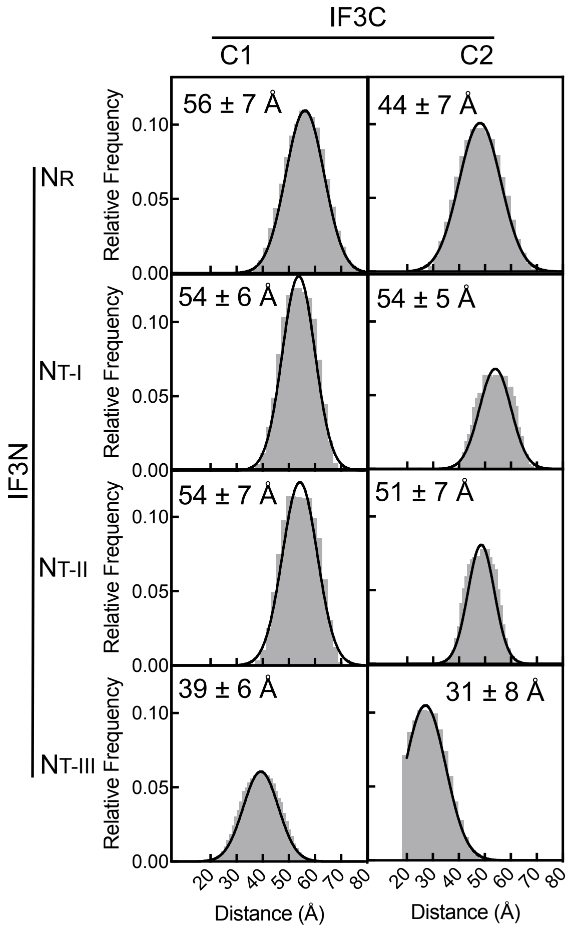

Supplementary figure 3. Structural analysis of IF3$_{DL}$ on 30S complexes. Frequency distribution of the distances between donor and acceptor dyes for IF3 domains occupying all combinations of binding sites. The median plus its deviation is indicated.

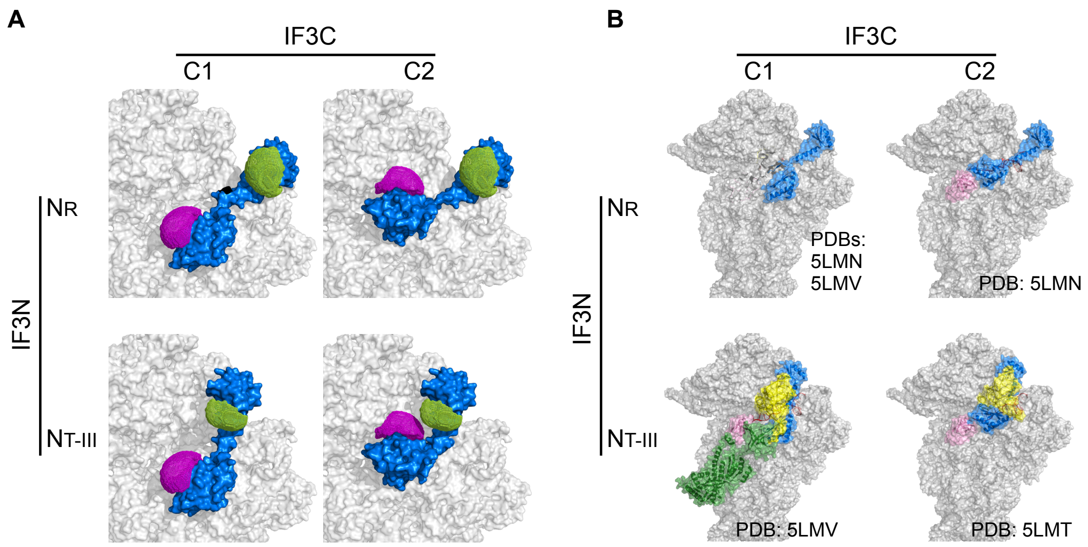

Supplementary figure 4. Structural representation of $IF3_{DL}$ on 30S complexes. (A) IF3 (blue) domains in the N_R, N_T, C_1, and C_2 binding configurations in the 30S platform. Donor (Alexa 488) accessible volume (AV) is shown in green and acceptor (Atto540Q) AV in magenta. The C1-N_R positioning, which has not been described in previous structural studies (22), derives after modeling IF3 in this work. All other three IF3 states are redrawn with the modeled dyes attached to the factor (see methods). (B) Structures of 30S initiation intermediates that correspond to IF3 positioning in (A) in the presence of ligands. IF3 (blue), IF1 (pink), fMet-tRNAfMet (yellow), and IF2 (green) are redrawn from the indicated PDBs (22).

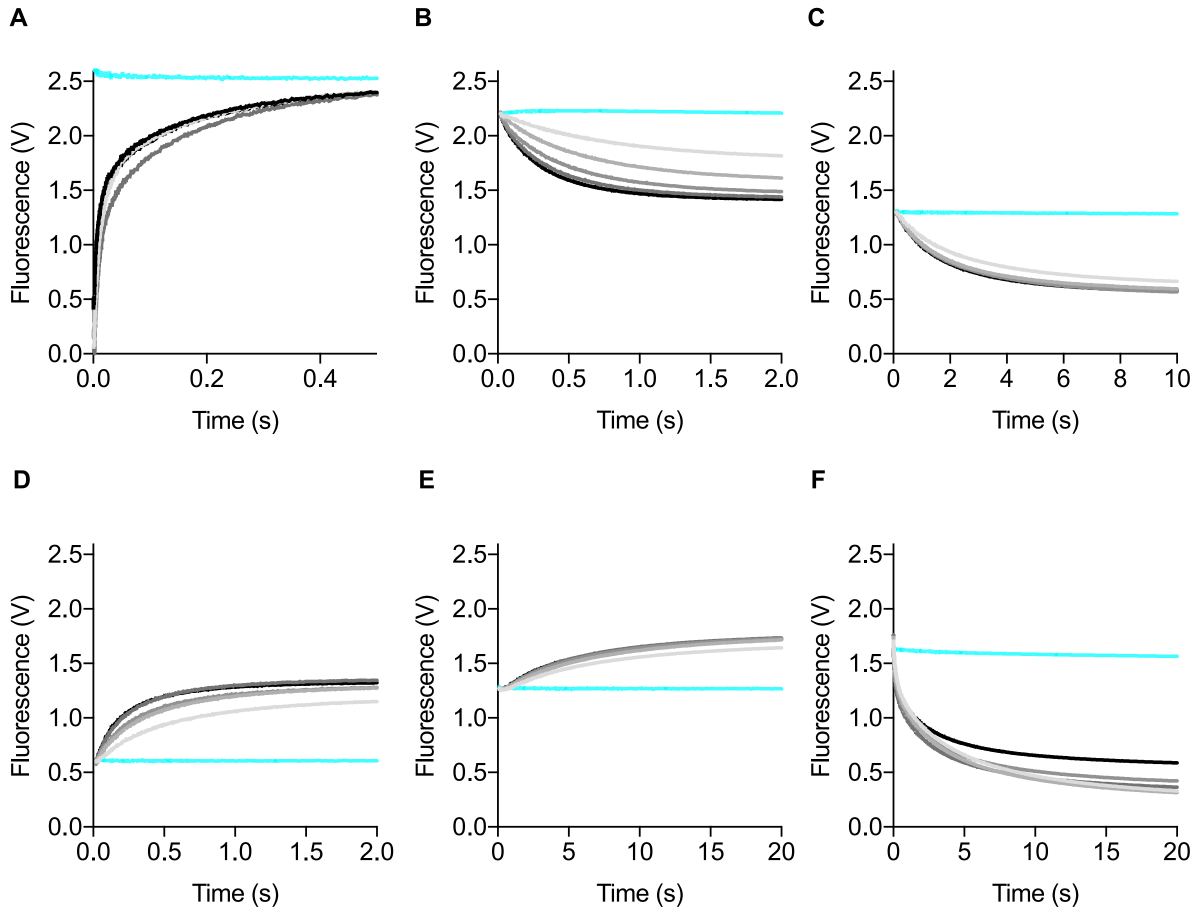

Supplementary figure 5. Time traces of $IF3_{DL}$ donor fluorescence change upon formation of all initiation intermediates. (A) Time courses of donor fluorescence change of 0.04 μM $IF3_{DL}$ during binding to varying concentration of 30S (0.15-0.5 μM). (B) Time courses of IF2 (0.15- 0.5 μM) binding to 0.05 μM 30S–$IF3_{DL}$ complexes. (C) Time courses of IF1 (0.15-0.5 μM) binding to 0.05 μM 30S–IF2–$IF3_{DL}$ complexes. (D) Time courses of fMet-$tRNA^{fMet}$ (0.15-0.5 μM) binding to 0.05 μM 30S–$IF3_{DL}$–IF1–IF2 complexes. (E) Time courses of mRNA (0.15-1 μM) binding to 0.05 μM 30S PICs ($IF3_{DL}$). (F) Time courses of 50S (0.15-1 μM) binding to 0.05 μM 30SIC ($IF3_{DL}$) complexes. A control against buffer is shown in each titration (sky blue). Each time trace results from the average of 5-7 independent measurement.

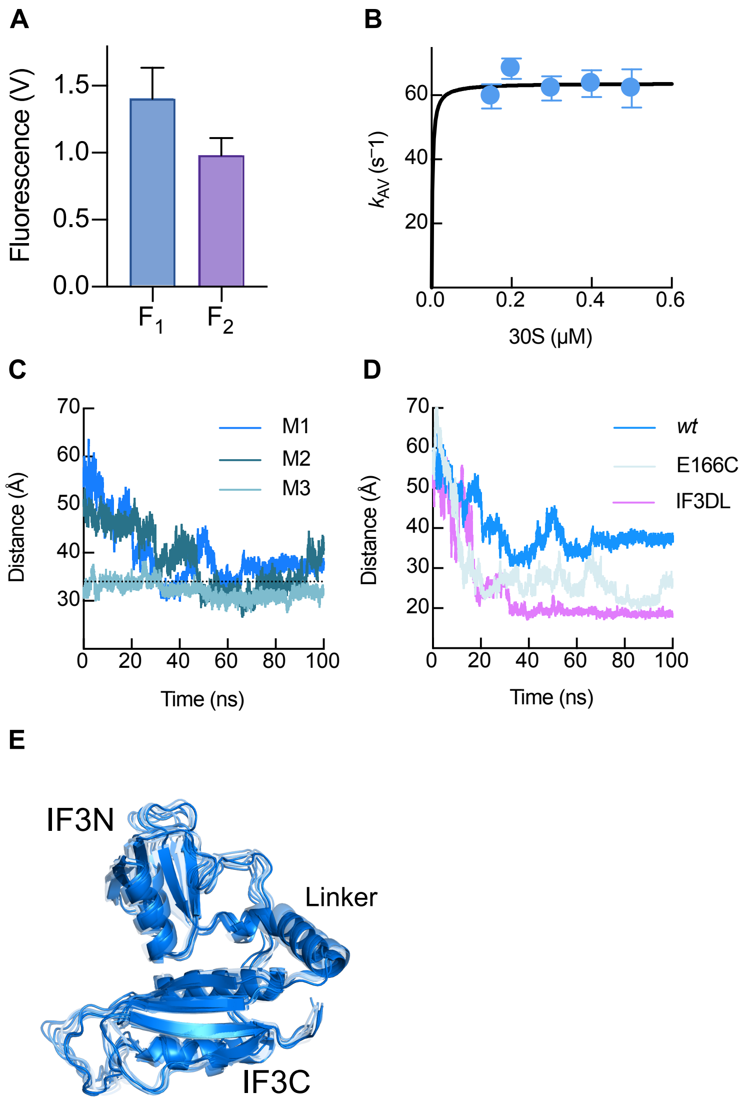

Supplementary figure 6 (Related to Figure 2). $IF3_{DL}$ binding to the 30S. (A) Average fluorescence change for each phase of the reaction, F_1 and F_2. (B) $IF3_{DL}$ average opening rates (k_{AV}) as a function of 30S concentration. 5-7 measurements for each 30S concentration were independently analyzed with equation 1. The resulting apparent rate replicates were used to calculate mean (blue circles) and standard deviations (error bars) using equation 3 and 4 (See Material and Methods). Continuous lines represent non-linear fitting with hyperbolic function (Equation 2). (C) Distance between residues 65 and 166 for MD trajectories of three different starting conformations of free IF3; in all three cases the distance converges to a range of 28-43 Å. (D) Distance between residues 65 and 166 for *wild type*, E166C, and labeled IF3 ($IF3_{DL}$) during MD simulation. For *wild type* IF3 the distance converges to the narrow range of 37-40 Å. (E) Compact conformations of IF3 reached after 100 ns of MD simulation.

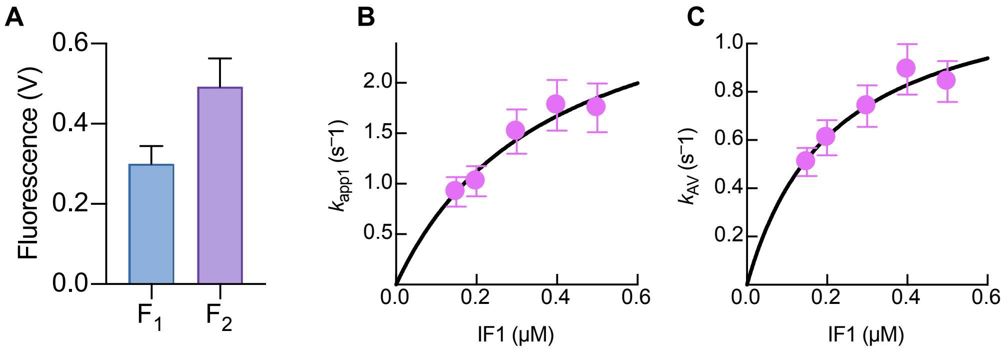

Supplementary figure 7 (Related to Figure 3). IF1 binding to the 30S-IF3$_{DL}$-IF2 complex. (A) Average fluorescence change for each phase of the reaction, F_1 and F_2. (B) IF1 concentration dependence of the k_{app1} of the fastest IF3$_{DL}$ closure reaction upon binding of the factor to the 30S–IF3$_{DL}$–IF2 complex. (C) IF3$_{DL}$ average closing rates (k_{AV}) as a function of IF1 concentration. 5-7 measurements for each IF1 concentration were independently analyzed with equation 1. The resulting apparent rate replicates were used to calculate mean (purple circles) and standard deviations (error bars) using equation 3 and 4 (See Material and Methods). Continuous lines represent non-linear fitting with a hyperbolic function (Equation 2).

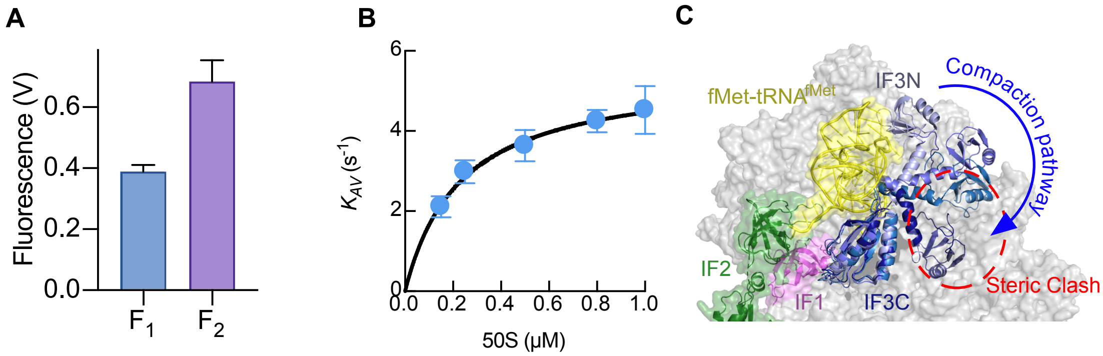

Supplementary figure 8 (Related to Figure 6). 50S subunit joining to the 30S-IC. (A) Average fluorescence change for each phase of the reaction, F_1 and F_2. (B) IF3$_{DL}$ average closing rates (k_{AV}) as a function of 50S concentration. 5-7 measurements for each 50S concentration were independently analysed with equation 1. The resulting apparent rate replicates were used to calculate mean (blue circles) and standard deviations (error bars) using equation 3 and 4 (See Material and Methods). Continuous line represents non-linear fitting with hyperbolic function (Equation 2). (C) Superimposing of the free IF3 trajectory provided by MD simulation over the 30S IC,

indicating a potential pathway that IF3 might follow to reach the compact conformation in the IC and during dissociation.

Supplementary Table 1. Distances between fluorophores and domains on $IF3_{DL}$ layouts.

30S complexes (this study)	Hussain et al nomenclature	Domain Position	Distance between dyes (Å)*	Distance measurements (N x 10^5)	Distance between aa 65-166 (Å)**	Distance between aa 38- 97 (Å)***
30S-IF3		$C_1 – N_R$	57 ± 7	15.6	68	73
30S-IF1-IF3	PIC1	$C_2 – N_R$	44 ± 7	14.9	56	72
30S-IFs		C_2 - N_{TIII}	31 ± 8	9.7	42	59
30S pre-IC	PIC2	C_2 - N_{TI}	54 ± 5	7.3	50	63
	PIC3	C_2 - N_{TII}	51 ± 5	6.2	45	59
30S IC		C_1 - N_{TII}	54 ± 6	9.9	68	69
30S IC/70S IC	PIC4	C_1 - N_{TIII}	39 ± 6	11.1	56	65
		C_1 - N_{TI}	54 ± 6	8.8	61	68

*Distances determined from the Fluorophores Accessible Volumes (See Methods).
**Distance between labelled cysteines in each static domain in the PDB files.
***Distance between labelled cysteines of the triple IF3 mutant used by Elvekrog & Gonzales, 2015 (21).

Supplementary Table 2. Calculated accessible volume (AV) for each fluorophore at all binding conformations of $IF3_{DL}$. AV spatial coordinates were generated with the FPS-software.

Site	AV pts	Volume (Å^3)*	AV pts (no tRNA)	Volume (Å^3)* (no tRNA)
C_2	4338	3805.82	4338	3805.82
C_1	4975	4364.68	4975	4364.68
N_R	3384	2968.86	3384	2968.86
N_{TI}	192	168.45	2341	2053.81
N_{TII}	216	189.50	2436	2137.16
N_{TIII}	223	195.64	2009	1762.54
Max	16114**	14137.17	16114**	14137.17

*Volume approximation by the number of coordinates to the AV to a sphere with a radius of 15 Å. **Maximum theoretical number of coordinates present in an AV with the following parameters: Linker length: 15 Å; Width: 4.5 Å; Dye radius: 4.5 Å.
